# Identification and Functional Characterization of Abiotic Stress Tolerance-Related *PLATZ* Transcription Factor Family in Barley (*Hordeum vulgare* L.)

**DOI:** 10.3390/ijms251810191

**Published:** 2024-09-23

**Authors:** Kangfeng Cai, Xiujuan Song, Wenhao Yue, Lei Liu, Fangying Ge, Junmei Wang

**Affiliations:** 1 Institute of Crop and Nuclear Technology Utilization, Zhejiang Academy of Agricultural Sciences, Hangzhou 310021, China; caikf@zaas.ac.cn (K.C.); 15168411002@163.com (X.S.); yuewh@zaas.ac.cn (W.Y.); liulei453@126.com (L.L.); 17855060921@163.com (F.G.); 2 National Barley Improvement Centre, Hangzhou 310021, China; 3 College of Advanced Agricultural Sciences, Zhejiang Agricultural and Forestry University, Hangzhou 311300, China

**Keywords:** PLATZ, transcription factor, barley, expression, abiotic stress response, functional characterization

## Abstract

Plant AT-rich sequence and zinc-binding proteins (PLATZs) are a novel category of plant-specific transcription factors involved in growth, development, and abiotic stress responses. However, the *PLATZ* gene family has not been identified in barley. In this study, a total of 11 *HvPLATZs* were identified in barley, and they were unevenly distributed on five of the seven chromosomes. The phylogenetic tree, incorporating PLATZs from *Arabidopsis*, rice, maize, wheat, and barley, could be classified into six clusters, in which HvPLATZs are absent in Cluster VI. *HvPLATZs* exhibited conserved motif arrangements with a characteristic PLATZ domain. Two segmental duplication events were observed among *HvPLATZs*. All *HvPLATZs* were core genes present in 20 genotypes of the barley pan-genome. The *HvPLATZ5* coding sequences were conserved among 20 barley genotypes, whereas *HvPLATZ4/9/10* exhibited synonymous single nucleotide polymorphisms (SNPs); the remaining ones showed nonsynonymous variations. The expression of *HvPLATZ2/3/8* was ubiquitous in various tissues, whereas *HvPLATZ7* appeared transcriptionally silent; the remaining genes displayed tissue-specific expression. The expression of *HvPLATZs* was modulated by salt stress, potassium deficiency, and osmotic stress, with response patterns being time-, tissue-, and stress type-dependent. The heterologous expression of *HvPLATZ3/5/6/8/9/10/11* in yeast enhanced tolerance to salt and osmotic stress, whereas the expression of *HvPLATZ2* compromised tolerance. These results advance our comprehension and facilitate further functional characterization of *HvPLATZs*.

## 1. Introduction

Transcription factors play critical roles in plant growth, development, and responses to external stimuli by activating or repressing target gene expression. Some transcription factors are ubiquitous in all eukaryotic organisms, while others are plant specific. In *Arabidopsis*, over 1500 transcription factors are encoded by >5% of its genome, with approximately 45% being plant specific [1].

PLATZs are plant-specific transcription factors and contain two conserved domains: C-x_2_-H-x_11_-C-x_2_-C-x_(4–5)_-C-x_2_-C-x_(3–7)_-H-x_2_-H and C-x_2_-C-x_(10–11)_-C-x_3_-C, and both are essential for zinc-dependent DNA binding. The first *PLATZ* gene was cloned from pea (*Pisum sativum*) in 2001 and was responsible for transcriptional repression mediated by an A/T-rich sequence [2]. Subsequently, more *PLATZ* genes were cloned and functionally identified in multiple plant species.

PLATZs regulate plant growth and development. *AtPLATZ1* (*ABA-INDUCED expression 1*, *AIN1*) affects abscisic acid (ABA)-inhibited elongation of the primary root by regulating reactive oxygen species (ROS) homeostasis [3]. *AtPLATZ3* (*ORESARA15, ORE15*) has been demonstrated to promote leaf growth by enhancing the rate and duration of cell proliferation during the early stage and restrains leaf senescence by regulating the GRF/GIF pathway during the later stage [4]. *AtPLATZ3* also regulates the size of the root apical meristem through the antagonistic interplay between auxin and cytokinin signaling-related pathways [5]. In rice, *GL6* positively regulates grain length by facilitating cell proliferation in developing panicles and grains [6]. Rice knockout lines of *OsFl3* exhibited a decrease in thousand-kernel weight and grain size [7]. *SG6* (*SHORT GRAIN6*) was also found to determine rice grain size through the division control of spikelet hull cells [8]. The interaction of maize *ZmPLATZ12* (*fl3*) with RPC53 and TFC1, which are crucial elements of the RNA polymerase III transcription complex, affects endosperm development and storage reserve filling [9,10]. *TaPLATZ-A1* in wheat is predominantly expressed in elongating stems and developing spikes and exhibits significant genetic interactions with the dwarfing gene *RHT1* on plant height regulation [11]. The *GmPLATZ* gene binds to the promoters of cyclin genes and *GmGA20OX*, activating their expression for cell proliferation and therefore modulating seed size and weight [12]. The transcriptional repressor RhPLATZ9 plays a crucial role in regulating senescence in rose flowers, and the RhWRKY33a-RhPLATZ9-RhRbohD regulatory module functions as a protective mechanism to maintain petal ROS homeostasis, counteracting premature senescence induced by aging and stress [13]. *PtaPLATZ18* has been evidenced to play a pivotal role in the regulation of growth and vascular tissue development in poplar [14].

PLATZs also play pivotal functions in plant abiotic stress responses. In *Arabidopsis*, *AtPLATZ1* and *AtPLATZ2* contribute to seed desiccation tolerance, and constitutive expression of *AtPLATZ1* confers partial desiccation tolerance on transgenic plants [15]. *AtPLATZ4* enhances drought tolerance by adjusting the expression of *PIP2;8* and ABA signaling-related genes [16]. However, AtPLATZ2, functioning redundantly with AtPLATZ7, compromises the salt tolerance of seedlings by directly inhibiting *CBL4/SOS3* and *CBL10/SCaBP8* genes [17]. GmPLATZ17 suppresses the transcription of *GmDREB5*, thereby impairing soybean drought tolerance [18]. *GmPLATZ1* is induced by drought, salinity, and ABA, and overexpression of *GmPLATZ1* in *Arabidopsis* delays germination under osmotic stress [19]. Overexpression of cotton *GhPLATZ1* in *Arabidopsis* improves tolerance to osmotic and salt stress during the germination and seedling stages [20]. Overexpression of *PhePLATZ1* in *Arabidopsis* enhances drought tolerance through osmotic regulation, improvement in water retention capacity, and mitigation of membrane and oxidative damage [21]. *SiPLATZ12* suppresses the expression of *SiNHX*, *SiCBL*, and *SiSOS* genes, resulting in impaired salt tolerance in foxtail millet [22]. *GhiPLATZ17* and *GhiPLATZ22* have been demonstrated to improve the salt tolerance of upland cotton [23]. Overexpression of *PtPLATZ3* maintains chlorophyll content stability and membrane integrity and induces the expression of genes associated with cadmium tolerance and accumulation, substantially enhancing cadmium tolerance and accumulation [24]. Additionally, transcriptome analyses have revealed the involvement of PLATZs in plant response to drought [25], heat [26], and hormones [27] across diverse plant species.

The PLATZ family has been identified in multiple plant species, including *Arabidopsis tahliana*, rice, maize [10], *Brassica rapa* [28], wheat [29], alfalfa [30], and buckwheat [31]. However, gene family identification has not been reported in barley. In this study, 11 *HvPLATZs* were identified in barley, and systemic in silico analyses were conducted. The response patterns of *HvPLATZs* to abiotic stresses in barley roots and leaves were examined, and their functions in salt and osmotic stress tolerance were characterized in yeast. These findings provide a foundation for further functional characterization of *HvPLATZs*.

## 2. Results

### 2.1. Identification of PLATZ Genes in Barley

In total, 11 *HvPLATZ* genes were identified in barley (Table 1). The protein lengths of *HvPLATZs* varied from 212 aas to 272 aas, with 1–3 introns. Their isoelectric points (pI) were 7.15–9.44 and theoretical MW was 22.74–29.21 kDa (Table 1). The instability indices ranged from 48.06 (HvPLATZ9) to 71.76 (HvPLATZ1), all >40, suggesting that these proteins are relatively unstable [32]. HvPLATZs were localized in the nucleus, except HvPLATZ4, which was localized in the extracellular space (Table 1).

### 2.2. Phylogeny and Gene Structure Analysis of HvPLATZs

**Figure 1:**
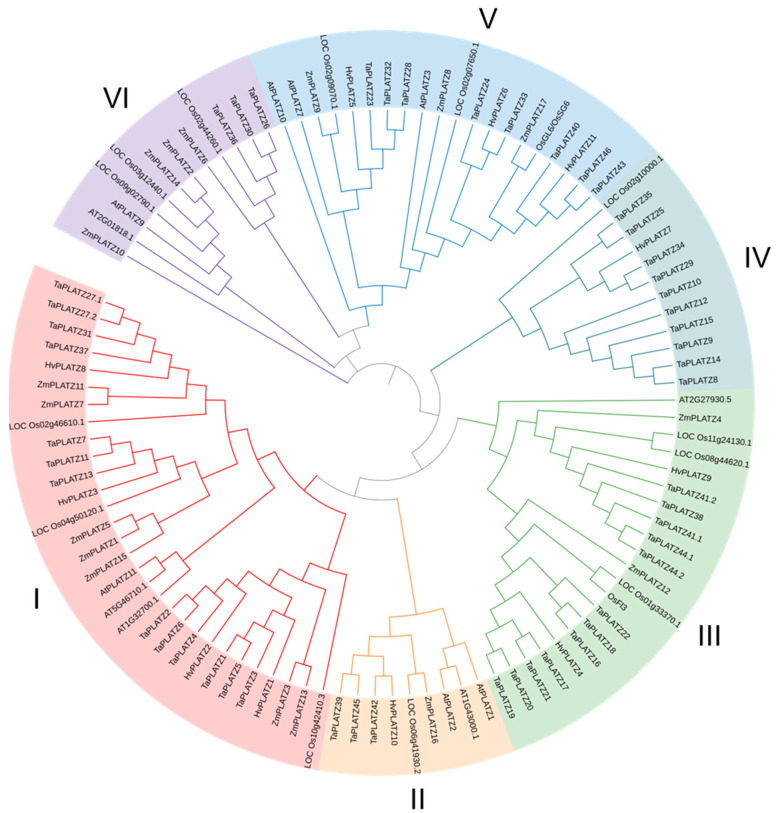
Phylogenetic tree of *PLATZ* family proteins from Arabidopsis, rice, maize, and barley. At: *Arabidopsis thaliana*; Os: *Oryza sativa*; Zm: *Zea mays*; Hv: *Hordeum vulgare*.

The protein sequences of 12 *PLATZs* in *Arabidopsis*, 15 *PLATZs* in rice, 17 *ZmPLATZs* in maize, 49 *TaPLATZs* in wheat, and 11 *HvPLATZs* in barley were used for phylogeny analysis (Figure 1). These 104 PLATZs were classified into six clusters based on phylogenetic relationships. Cluster I had 30 members, followed by Cluster III and V, containing 21 and 20 members, respectively. Cluster IV and VI each contained 12 members, and only 9 members were classified in Cluster II. Clusters I to V contained four, one, two, one, and three *HvPLATZs*, respectively. Notably, no *HvPLATZ* was classified into Cluster VI in this phylogenetic tree; neither *AtPLATZ* nor *ZmPLATZ* was classified into Cluster IV.

**Figure 2:**
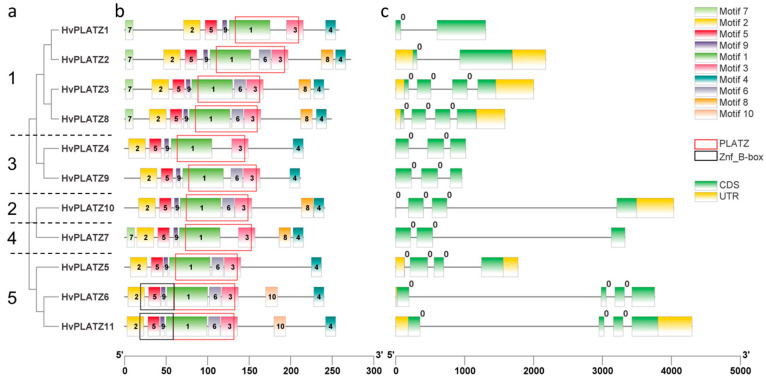
Phylogenetic relationship and sequence characteristics of *HvPLATZs*. (**a**) Phylogenetic tree of *HvPLATZs*, the *HvPLATZs* from Cluster I–V were designated as numbers 1–5, respectively; (**b**) conserved motifs of *HvPLATZs*; (**c**) gene structures of *HvPLATZs*.

*HvPLATZs* in the same cluster displayed similar motif arrangements (Figure 2). *HvPLATZ6* and *HvPLATZ11*, which possessed a B-box-type zinc finger domain (IPR000315, Znf_B-box), were in Cluster V and showed the same motif arrangements. According to the position between or within codons, introns are classified into three phases: phase 0, phase 1, and phase 2. Introns of phase 0 are located between two codons, whereas introns of phase 1 and phase 2 are located after the first position and after the second position in a codon, respectively [33]. Introns existed in all *HvPLATZ* genes, and intriguingly, all introns belonged to phase 0.

### 2.3. Chromosomal Distribution and Duplication of HvPLATZs

**Figure 3:**
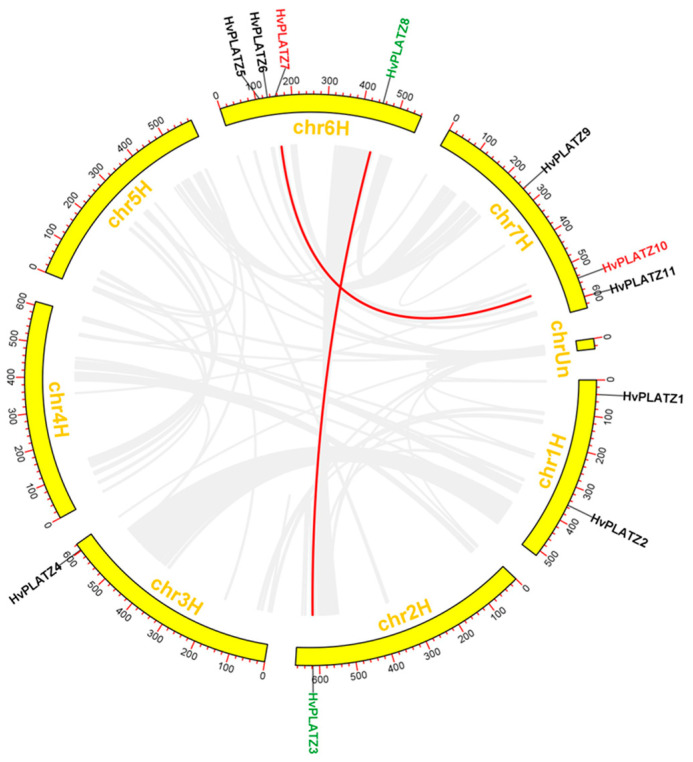
Chromosomal distribution and segmental duplication of *HvPLATZs*. Segmentally duplicated genes were displayed in the same color and linked with red lines.

The distribution of 11 *HvPLATZs* was uneven across five out of seven chromosomes, with four genes on chromosome 6, three genes on chromosome 7, two genes on chromosome 1, and one gene each on chromosomes 2 and 3 (Figure 3). No *HvPLATZ* genes were found on chromosomes 4 and 5.

Gene duplication is recognized as the major driving force behind gene family expansion during evolution [34]. The *HvPLATZ* gene family did not exhibit tandem duplication events. In total, two pairs of *HvPLATZ* genes were segmentally duplicated (*HvPLATZ3* and *HvPLATZ8*; *HvPLATZ7* and *HvPLATZ10*; Figure 3).

### 2.4. Cis-Acting Elements Identification

**Figure 4:**
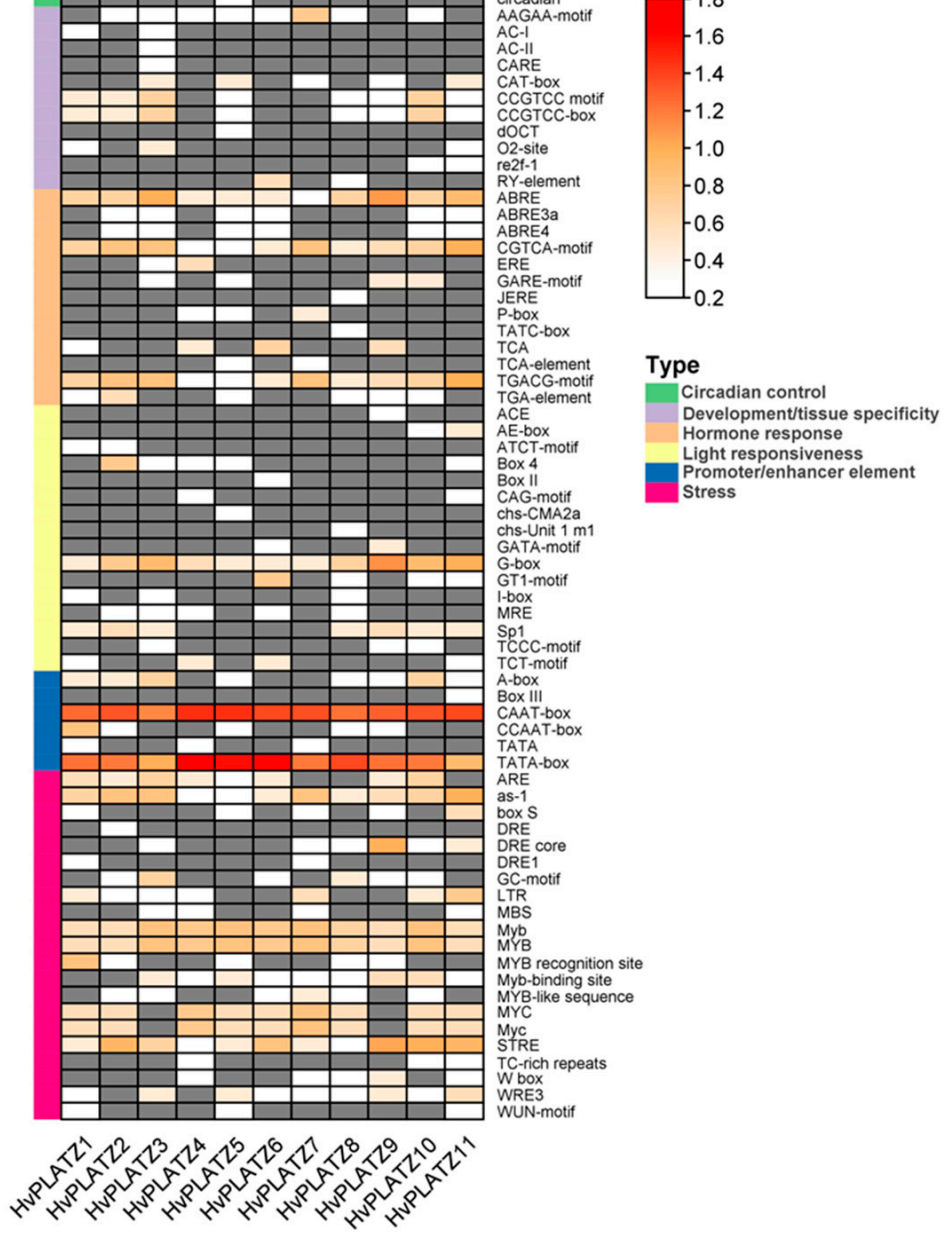
*Cis*-acting elements in promoters of *HvPLATZs*. *Cis*-acting elements were predicted following the 2-kb sequences upstream of the coding sequences of *HvPLATZs*. The quantity of *cis*-acting elements was normalized by log_10_(number + 1) and then used for visualization. Absent elements were displayed in grey color.

Sixty-eight *cis*-acting elements were identified and could be sorted into six functional types (Figure 4; File S1). Approximately one-third (21, 30.9%) of the elements were classified as “stress” responsive, followed by those involved in “light responsiveness” (16, 23.5%), “hormone response” (13, 23.5%), “development/tissue specificity” (11, 16.2%), and “promoter/enhancer element” (6, 8.8%). Only one type of element was observed in the “circadian control” category (Figure 4). In the “promoter/enhancer element” category, CAAT-box and TATA-box, which are binding sites of RNA polymerase and contribute to transcription efficiency, were present in all *HvPLATZ* promoters, suggesting their important roles in regulating *HvPLATZs* transcription. In the “hormone response” category, ABRE is involved in ABA responsiveness, while the CGTCA-motif and TGACG-motif were related to methyl jasmonate responsiveness, all of which widely existed in *HvPLATZ* promoters (Figure 4). In the “light responsiveness” category, G-box was also extensively present in promoters of *HvPLATZs* (Figure 4). Moreover, stress-responsive motifs, such as as-1, Myb, MYB, and STRE, were ubiquitously detected in all *HvPLATZs*. However, other *cis*-acting elements were relatively gene-specific and low-abundance. Therefore, *HvPLATZs* likely play crucial roles in hormone and stress responses but might vary in their response patterns and expression profiles.

### 2.5. Syntenic Analysis of HvPLATZs

**Figure 5:**
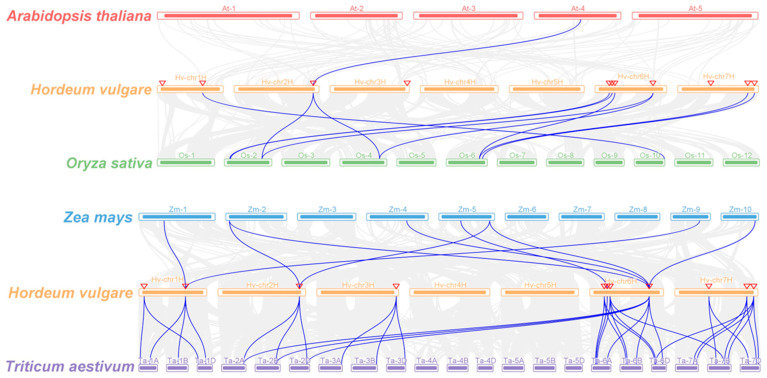
Synteny analyses of PLATZs between barley and four plant species (*Arabidopsis thaliana*, *Oryza sativa*, *Zea mays*, and *Triticum aestivum*). Gray lines indicated collinear blocks and blue lines highlighted syntenic *PLATZs* gene pairs. Inverted triangles indicated the chromosomal positions of *HvPLATZs*.

The synteny of *PLATZs* between barley and other plant species was analyzed (Figure 5; File S2). Among these comparisons, *HvPLATZ3* and *AtPLATZ11* were the only pair of orthologs detected between barley and *Arabidopsis*. The comparison between barley and rice revealed the presence of ten pairs of orthologous genes, comprising eight *HvPLATZs* in barley and seven corresponding genes in rice. *HvPLATZ3* and *HvPLATZ8* exhibited orthology to two *PLATZ* genes in rice (*LOC_Os02g46610* and *LOC_Os04g50120*), while *HvPLATZ7* and *HvPLATZ10* were orthologous to *LOC_Os06g41930*. The comparison between maize and barley revealed the presence of nine pairs of orthologous genes, including seven *ZmPLATZs* and four *HvPLATZs*. *HvPLATZ2* and *HvPLATZ3* were identified as orthologs of two *ZmPLATZ* genes, namely *ZmPLATZ3/13* and *ZmPLATZ5/11*, respectively. Notably, *HvPLATZ8* was orthologous to four *ZmPLATZs* (*ZmPLATZ5/7/11/15*). The evolutionary proximity between barley and wheat resulted in the detection of substantially more orthologous gene pairs (34 pairs, including all *HvPLATZs* and 30 *TaPLATZs*) between these two species than those between rice and maize (Figure 5; File S2). *HvPLATZ8* was orthologous to six *TaPLATZs*, followed by *HvPLATZ7/11* and *HvPLATZ2/3/4/5/10*, which were orthologous to four and three *TaPLATZs*, respectively. *HvPLATZ1/9* and *HvPLATZ6* were orthologous to two and one *TaPLATZ*, respectively.

### 2.6. Natural Variation of HvPLATZs in Barley

**Figure 6:**
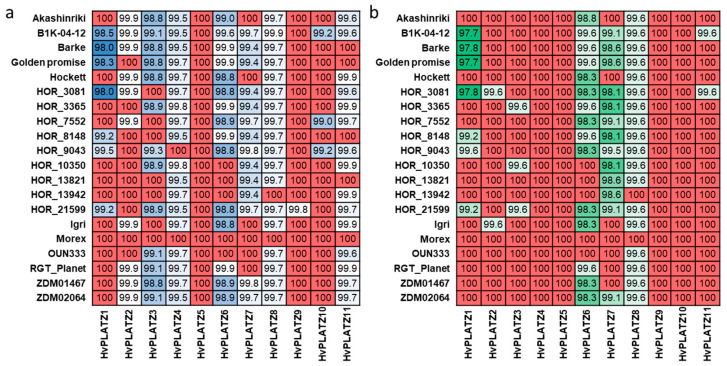
Nucleotide (**a**) and amino acid (**b**) sequence identity of *HvPLATZs* among barley genotypes. Sequences of *HvPLATZs* in Morex were used as Blast queries, and the percent identity was designated in grids. Red color indicated absolute identity, and the darker of blue and green, the higher of nucleotide and amino acid identity, respectively.

A pan-genome refers to the diversity within a species in terms of genome content and structure [35]. Natural variation of genes in a pan-genome context includes not only genic presence/absence variation (PAV), but also base-level SNPs [36,37]. To investigate the natural variation of *HvPLATZs*, PAV and SNP were examined on the basis of the first generation of the barley pan-genome (Figure 6; Files S5 and S6) [36]. Notably, the coding sequences of *HvPLATZ5* and *HvPLATZ10* in assemblies of 17 genotypes featured CAT as start codons instead of the typical ATG (File S3). However, in de novo assemblies, the start codon was the typical ATG (File S3). Therefore, the discrepancy was probably attributable to the assembly process, and the subsequent analyses were conducted using sequences in which the initial codon CAT was replaced with the typical start codon ATG (File S4). The pan-genome can be classified into two categories: the core genome and the dispensable genome. The former consists of genes that are universally present across all genotypes, whereas the latter consists of genes that are absent in certain genotypes [35,38]. The 11 *HvPLATZs* were all a part of the core genome, accordingly (Figure 6). The nucleotide and protein sequences of *HvPLATZs* were identical in two to eight and five to ten genotypes, respectively, when compared with the reference cultivar Morex. Furthermore, the coding and protein sequence identity of *HvPLATZs* between Morex and other barley genotypes ranged from 98.0% to 100% and from 97.7% to 100%, respectively. The coding sequence of *HvPLATZ5* was identical among all 20 genotypes, followed by *HvPLATZ9* and *HvPLATZ10*, which shared identity among 19 and 17 genotypes, respectively. The coding sequences of *HvPLATZ4* and *HvPLATZ8* in eighteen genotypes exhibited SNPs compared with Morex. However, the SNPs in *HvPLATZ4* were synonymous, whereas those in *HvPLATZ8* were nonsynonymous. In addition to *HvPLATZ5*, the protein sequences of *HvPLATZ4*, *HvPLATZ9*, and *HvPLATZ10* were identical across all 20 genotypes. Moreover, the wild barley genotype, B1K-04-12, exhibited the most *HvPLATZ*s with SNPs compared with Morex. HOR_3081 possessed the most *HvPLATZ* members, with amino acid sequences distinct from those of Morex.

### 2.7. Tissue-Specific Expression of HvPLATZs

**Figure 7:**
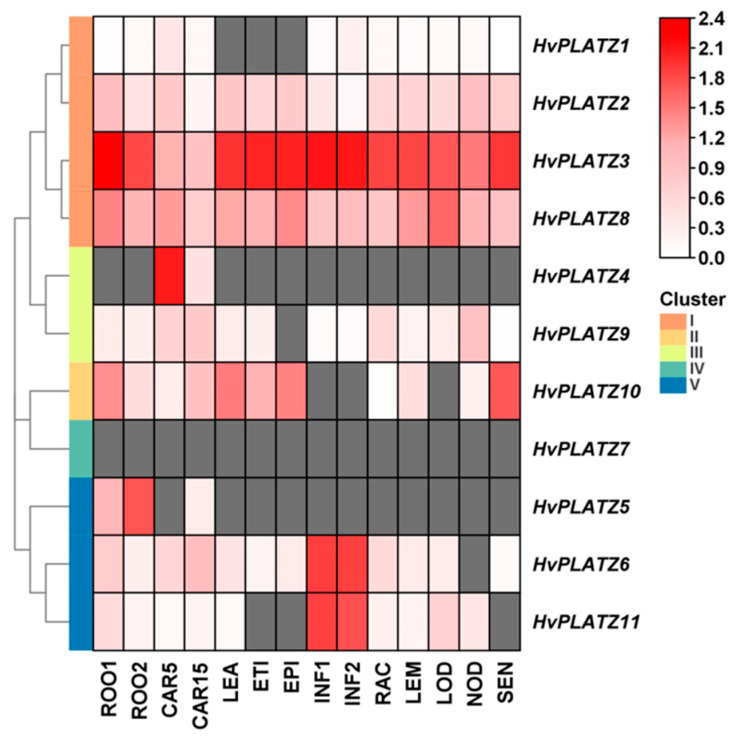
Expression profiling of *HvPLATZs* in 14 tissues following transcriptomic data. FPKM values were normalized using log_10_(FPKM + 1) transformation. Grids filled with grey color indicated the absence of detectable gene expression. ROO1, roots from seedlings (10 cm shoot stage); ROO2, roots (28 DAP); CAR5, developing grain (5 DAP); CAR15, developing grain (15 DAP); LEA, shoots from seedlings (10 cm shoot stage); ETI, etiolated seedling, dark condition (10 DAP); EPI, epidermal strips (28 DAP); INF1, young developing inflorescences (5 mm); INF2, developing inflorescences (1–1.5 cm); RAC, inflorescences, rachis (35 DAP); LEM, inflorescences, lemma (42 DAP); LOD, inflorescences, lodicule (42 DAP); NOD, developing tillers, 3rd internode (42 DAP); SEN, senescing leaves (56 DAP).

The tissue expression patterns of *HvPLATZs* were further analyzed (Figure 7; File S5). *HvPLATZ2/3/8* in Cluster I expressed ubiquitously across all 14 tissues and stages, with exceptionally high levels for *HvPLATZ3*, suggesting critical involvement in growth and regulation. However, the expression of *HvPLATZ7* from Cluster IV was not detected in any tested tissues, implying that these genes may be redundant or silent. The expression of the remaining seven *HvPLATZ*s was detected in at least two tissues, exhibiting tissue-specific patterns of expression. Furthermore, *HvPLATZ5* was not detected in developing grain at 5 d after pollination but exhibited expression at 15 d after pollination. Therefore, the expression of *HvPLATZs* is tissue-specific and developmental stage-dependent.

### 2.8. Transcriptional Responses of HvPLATZs to Abiotic Stresses

**Figure 8:**
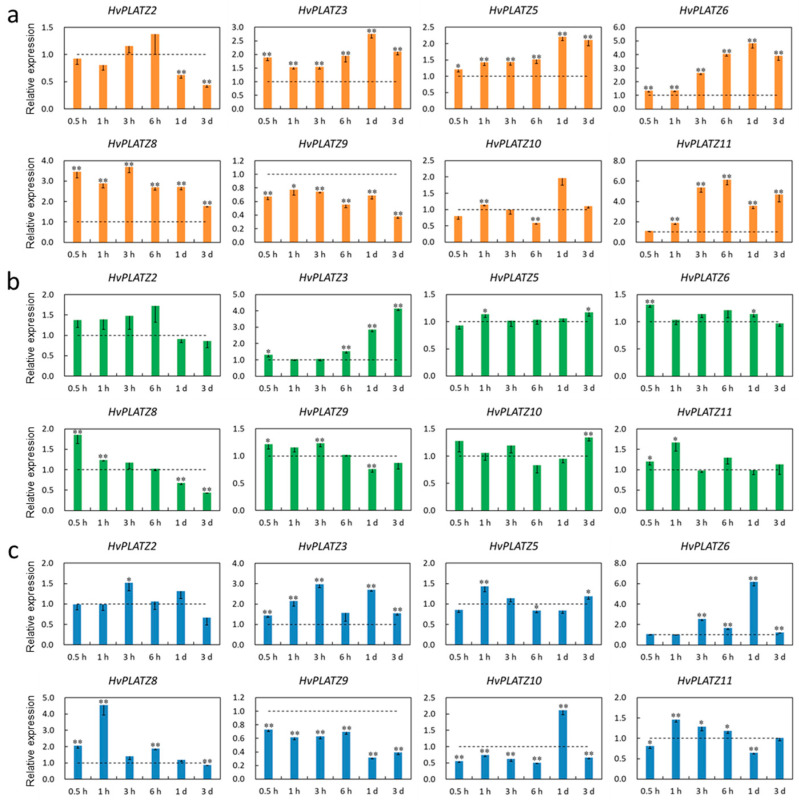
Expression levels of *HvPLATZs* in barley roots in response to salt stress (**a**), potassium deficiency (**b**), and osmotic stress (**c**) at the seedling stage. Dotted lines indicated the expression levels of *HvPLATZs* in control seedlings. * and ** indicate significant difference at *p* < 0.05 and *p* < 0.01, respectively.

The transcriptional responses of *HvPLATZs* to salt, potassium deficiency, and osmotic stress treatments were investigated in roots and leaves. The expression of *HvPLATZ1/4/7* was not detectable in roots under both control and stress conditions; therefore, only the results of the other eight *HvPLATZs* were presented (Figure 8). *HvPLATZs* were significantly induced or suppressed after stress treatments, except for *HvPLATZ2* under low-potassium conditions (induced but not significantly). The expression of *HvPLATZ3/5/6/8* and *HvPLATZ9* was rapidly induced and suppressed, respectively, within 0.5 h of treatment initiation and throughout the entire treatment period. However, the expression of *HvPLATZ2/10* exhibited a dynamic pattern, alternating between induction and suppression (Figure 8a). The expression of *HvPLATZs* was relatively less affected by potassium deficiency compared with salt stress (Figure 8b). Notably, the expression of *HvPLATZ3* remained relatively stable after short-term (0.5–3 h) low-potassium treatment but was gradually and significantly induced via long-term (from 6 h to 3 d) treatment. The expression of *HvPLATZ8* was rapidly and considerably induced at the initiation of therapy but subsequently declined and became significantly suppressed during the later stage of the treatment (1–3 d). The expression of *HvPLATZ3* and *HvPLATZ9* was consistently induced and suppressed, respectively, under osmotic stress (Figure 8c). However, the expression of the remaining *HvPLATZs* fluctuated, alternating between induction and suppression.

**Figure 9:**
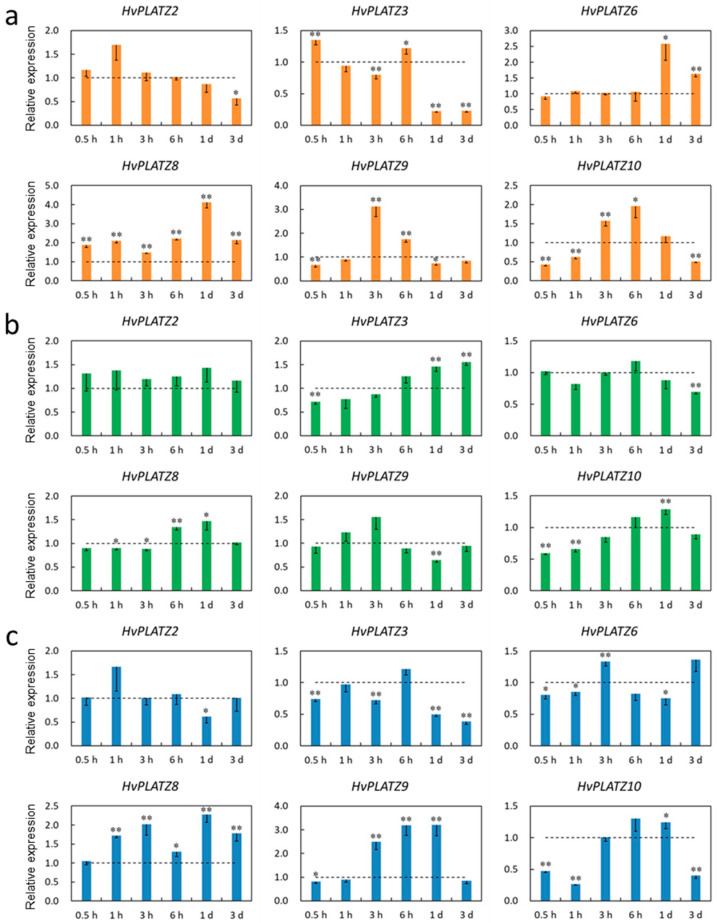
Expression levels of *HvPLATZs* in barley leaves in response to salt stress (**a**), potassium deficiency (**b**), and osmotic stress (**c**) at the seedling stage. Dotted lines indicated the expression levels of *HvPLATZs* in control seedlings. * and ** indicate significant difference at *p* < 0.05 and *p* < 0.01, respectively.

Only six *HvPLATZs* were detected in leaves via qRT-PCR (Figure 9). Only *HvPLATZ8* was significantly induced after salt stress treatment (Figure 9a). The induction of *HvPLATZ2* was consistently observed under potassium deficiency conditions, although the induction was not statistically significant (Figure 9b). After osmotic stress treatment for 1 h, the expression of *HvPLATZ8* was also significantly upregulated (Figure 9c). The expression of *HvPLATZs* exhibited pleiotropic and dynamic changes between induction and suppression, excluding the aforementioned cases (Figure 9), suggesting a more intricate regulatory mechanism for abiotic stresses in leaves than that in roots.

### 2.9. Functional Characterization of HvPLATZs in Yeast

**Figure 10:**
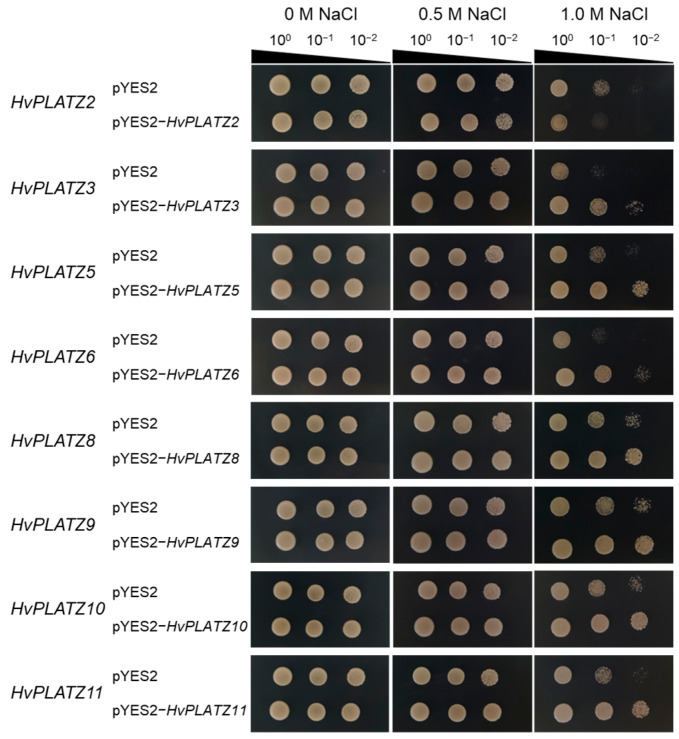
Influence of *HvPLATZs* expression in the INVSC1 yeast strain on salt stress tolerance. *HvPLATZs* were recombined into pYES2-NTB vectors, and the recombinant vectors were transformed into INVSC1 yeast strain, respectively. The transformed INVSC1 strains were cultured on SD-Ura medium for 2 d. Then PCR-verified single colonies were resuspended in sterile water, serially diluted, and cultured on SG-Ura media with different concentrations of NaCl for 5 d.

**Figure 11:**
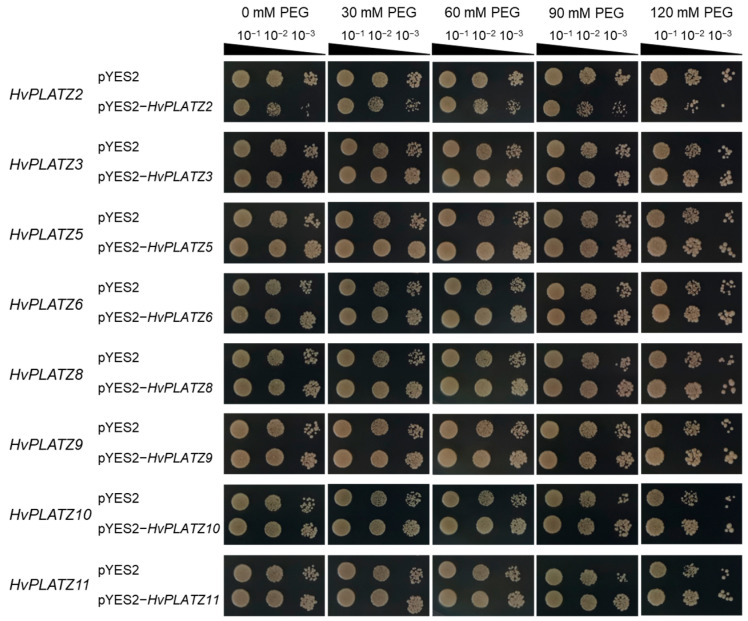
Influence of *HvPLATZs* expression in the INVSC1 yeast strain on osmotic stress tolerance. *HvPLATZs* were recombined into pYES2-NTB vectors, and the recombinant vectors were transformed into INVSC1 yeast strain, respectively. The transformed INVSC1 strains were cultured on SD-Ura medium for 2 d. Then PCR-verified single colonies were resuspended in sterile water and shaken cultured on SG-Ura media containing various concentrations of PEG3350 for 3 d. Yeast solutions were serially diluted and cultured on SG-Ura media for 4 d.

The qRT-PCR results indicated the expression of eight *HvPLATZs* in barley roots, with diverse responses to abiotic stresses (Figure 8 and Figure 9). To validate their roles in salt and osmotic stress responses, these genes were functionally characterized in yeast (Figure 10 and Figure 11). The yeast strains expressing both empty vector (pYES2) and recombinant vectors (pYES2-*HvPLATZs*) exhibited robust growth under normal conditions, performing similarly across various dilutions (Figure 10). However, growth differences were observed between strains expressing empty and recombinant vectors as the NaCl concentration in media increased to 1.0 M. The growth of strains expressing *HvPLATZ2* was inferior to the control, whereas those expressing *HvPLATZ3/5/6/8/9/10/11* exhibited superior growth compared with the control. These results revealed that *HvPLATZ2* negatively regulated salt tolerance in yeast, whereas *HvPLATZ3/5/6/8/9/10/11* positively regulated salt tolerance. In the osmotic stress tolerance essay, the robustness of strains decreased with increased dilution under control and osmotic stress conditions (Figure 11). Heterologous expression of *HvPLATZ2* led to a decrease in osmotic stress tolerance in recombinant yeast strains, whereas the expression of *HvPLATZ3/5/6/8/9/10/11* enhanced osmotic stress tolerance, consistent with the results observed in the salt tolerance assay (Figure 10).

## 3. Discussion

### 3.1. PLATZ Family in Different Plant Species

*TaPLATZ* family members were identified as 49 and 62 by He et al. [29] and Fu et al. [39], respectively, with this discrepancy due to the inclusion of 13 additional scaffolds in the latter research. The current analyses involving PLATZs from wheat were based on the 49 *TaPLATZs* identified by He et al. [29]. Whole-genome duplication or polyploidization, tandem and segmental duplication are primary driving forces for gene family expansion [40]. The PLATZ members in *Arabidopsis*, rice, maize, and barley were 12, 15, 17, and 11, respectively, despite significant variations in genome sizes ranging from ~135 Mb (*Arabidopsis*) to ~5.3 Gb (barley). Thus, the number of PLATZ members in these species appeared to be relatively conserved. As an allohexaploid with a genome size of ~17 Gb, the wheat genome contained 49 *TaPLATZs*, triple the number of *PLATZs* in rice and maize, implying that polyploidization was the major driving force of *PLATZ* family expansion at a species level. Wheat shared the closest evolutionary relationship with barley in this study, which was reflected in phylogenetic and syntenic analyses (Figure 1 and Figure 5). The wheat genome exhibited nine tandem duplication events involving 20 *TaPLATZs*, while the barley genome displayed two segmental duplication events involving four *HvPLATZs* (Figure 3). These findings suggested that wheat and barley employed distinct strategies in expanding their *PLATZ* gene families.

HvPLATZ6 and HvPLATZ11 contained an additional Znf_B-box, similar to results reported in *PLATZ* families from maize [10], *Brassica rapa* [28], alfalfa [30], and tomato [41]. Deletion of the Znf_B-box led to a significant reduction in the anti-stress activities of MdBBX10 in *E. coli*, whereas the segment containing the Znf_B-box retained more than half of the activities of MdBBX10, suggesting that the Znf_B-box played a crucial role in stress tolerance [42]. The robust activities of *HvPLATZ6/11* in the salt stress response in roots might be partially attributed to the presence of a Znf_B-box (Figure 8).

The peptide length of *HvPLATZs* ranged from 212 to 272 aas, similar to that of *AtPLATZs* from Arabidopsis (110–256 aas), *OsPLATZs* from rice (171–298 aas, except *LOC_Os11g24130.1*), *ZmPLATZs* from maize (198–309 aas), and *TaPLATZs* from wheat (158–275 aas). Although the peptide length of *LOC_Os11g24130.1* was merely 80 aas, it possessed an intact PLATZ domain. Notably, the peptide length of *MbPLATZ6* and *MbPLATZ16* from *M. baccata* [43], as well as *MsPLATZ17* from *Medicago sativa* [30] was 892 aas, 737 aas, and 629 aas, respectively. The anomalous phenomena might be ascribed to the inaccuracy in genome assembly and annotation, necessitating further analyses for validation. The pI of certain PLATZ members from Arabidopsis, rice, maize [44], and wheat [29] was <7.0. Conversely, all HvPLATZ members from barley exhibited the pI of >7.0 (Table 1), indicating that all HvPLATZs were basic proteins. Similar results were also reported in the PLATZ families of buckwheat [31], watermelon [45], apple [46], *Populus trichocarpa* [24], and ginkgo [47], with pI higher than 7.0.

### 3.2. Expression of HvPLATZs in Different Tissues and Response to Abiotic Stresses

The expression of *HvPLATZ2/3/8* from Cluster I was detected in all 14 tested tissues (Figure 7), suggesting that *HvPLATZ2/3/8* may be housekeeping genes playing pivotal roles in growth and development. HvPLATZ7 appeared to be a silent gene with no detectable expression in any tested tissues or developmental stages (Figure 7). The remaining seven *HvPLATZs* exhibited tissue-specific and/or developmental stage-dependent expression. Notably, the *HvPLATZ* gene family in barley exhibited segmental duplication events involving two pairs of *HvPLATZs* (*HvPLATZ3/8* and *HvPLATZ7/10*, Figure 3). The segmentally duplicated gene pairs were phylogenetically clustered together (Figure 2), and the expression levels of *HvPLATZ3* and *HvPLATZ8* were comparably high and observed in all tested tissues and stages. However, *HvPLATZ10* was detected in 11 tissues and stages, whereas *HvPLATZ7* was completely absent in the detected tissues and stages (Figure 7). A similar phenomenon was also observed in poplar with segmentally duplicated *PagPLATZs* [48]. Additionally, *HvPLATZ3* and *HvPLATZ8* exhibited identical motif arrangements and introns, both classified into Cluster I. Conversely, *HvPLATZ7* and *HvPLATZ10* displayed divergent motif arrangements and introns, classified into Cluster IV and Cluster II, respectively (Figure 2). These results suggested that *HvPLATZ3* and *HvPLATZ8* were relatively conserved in terms of gene sequence, structure, and expression patterns. However, certain mutations might occur in *HvPLATZ7* or *HvPLATZ10* after segmental duplication, resulting in divergence in expression profiles and functions.

*Cis*-acting elements were abundantly detected in *HvPLATZ* promoters, incorporating 13 phytohormone-responsive elements and 21 stress-responsive ones (Figure 4; File S1). *Cis*-acting elements relevant to ABA and methyl jasmonate responsiveness, such as ABRE, CGTCA-motif, and TGACG-motif, as well as stress response-related elements like as-1, Myb, MYB, and STRE [49], were ubiquitously present in promoters of *HvPLATZs*. Therefore, the expression of *HvPLATZs* under abiotic stress conditions was determined (Figure 8 and Figure 9). The expression of eight *HvPLATZs* was detected in roots, whereas the expression of *HvPLATZ5* and *HvPLATZ11* was not observed in leaves (Figure 8 and Figure 9). The absence of *HvPLATZ5* expression in leaves, as confirmed with qRT-PCR, was consistent with transcriptomic data [50] obtained from shoots of seedlings at the 10 cm shoot stage (Figure 7). The expression of *HvPLATZs* was influenced by salt, potassium deficiency, and osmotic stress treatments, but the response patterns varied depending on stress types. For example, *HvPLATZ9* in roots was significantly downregulated under salt and osmotic stress conditions throughout the experiment, whereas it was upregulated after short-term (from 0.5 h to 3 h) and downregulated after long-term (from 1 to 3 d) potassium deficiency treatments, respectively. Similar results were also reported in *SlPLATZs* from tomato [51]. Moreover, tissue type also influenced response patterns. Under salt conditions, the expression of *HvPLATZ9* was significantly downregulated in roots, whereas it was significantly upregulated in leaves after treatment for 3 h and 6 h (Figure 8 and Figure 9). Irrespective of stress and tissue types, the response patterns of *HvPLATZs* to abiotic stresses could be classified into three groups: (1) induction throughout the entire period, such as *HvPLATZ3/5/6/8/11* in roots under salt conditions, *HvPLATZ3* in roots under osmotic stress conditions, and *HvPLATZ8* in leaves under salt and osmotic stress conditions; (2) suppression throughout the entire period, including *HvPLATZ9* in roots under salt and osmotic stress conditions; (3) dynamic oscillation between induction and suppression, such as *HvPLATZ10* in roots under osmotic stress conditions, *HvPLATZ3/10* in leaves under salt conditions, and *HvPLATZ10* in leaves under potassium deficiency conditions. These suggest that expression patterns of *HvPLATZs* were not only stress and tissue-type-related but also time-dependent.

### 3.3. Functions of HvPLATZs in Abiotic Stress Tolerance

The functional characterization of *HvPLATZs* in yeast revealed their involvement in salt and osmotic stress tolerance (Figure 10 and Figure 11). *HvPLATZ2* expression compromised yeast growth under these stress conditions, indicating a negative regulatory role in salt and osmotic stress tolerance. This is consistent with the functions of *AtPLATZ2/7* [17] and *SiPLATZ12* [22] in salt tolerance and *GmPLATZ17* [18] in drought tolerance. Conversely, the expression of *HvPLATZ3/5/6/8/9/10/11* improved yeast growth under salt and osmotic stress conditions, suggesting a positive role in salt and osmotic stress tolerance. These findings are consistent with the functions observed for *GhiPLATZ1/17/22* [20,23] in salt stress tolerance, *AtPLATZ4* [16] and *PhePLATZ1* [21] in drought tolerance, and *AtPLATZ1/2* [15] in seed desiccation tolerance. Additionally, some *PtPLATZs* from *Populus trichocarpa* have also been reported to enhance yeast tolerance to cadmium [24].

## 4. Materials and Methods

### 4.1. HvPLATZ Genes Identification

The protein sequences of *PLATZ* genes from Arabidopsis and rice were retrieved from TAIR (https://www.arabidopsis.org/, accessed on 17 April 2023) and RiceData (https://ricedata.cn/, accessed on 17 April 2023), respectively, and were used for a blast search against the barley genome database (Morex v3). Meanwhile, “IPR006734” was used to search against the barley genome in Ensembl Plants (http://plants.ensembl.org/, accessed on 17 April 2023). Furthermore, “PLATZ” was used as a keyword to search against barley annotation files. The putative *HvPLATZ* genes were further scanned in InterPro [52], and only those containing the complete “IPR006734” domain were retained. Eleven *HvPLATZ* genes were identified in the end.

### 4.2. Physicochemical Properties and Subcellular Localizations

The molecular weights (MW), isoelectric points (pI), and instability indices of HvPLATZs were predicted using ExPASy [53]. Subcellular localizations of HvPLATZs were predicted using BUSCA [54].

### 4.3. Phylogeny, Syntenic Relationship, and Duplication Analyses

The PLATZ protein sequences from *Arabidopsis* (12), rice (15), maize (17) [10], and wheat (49) [29] were retrieved from TAIR, RGAP, MaizeGDB, and EnsemblPlants, respectively. Sequences of barley and these four plants were aligned using MAFFT (https://www.ebi.ac.uk/jdispatcher/msa/mafft?stype=protein, accessed on 28 April 2023) [55], and phylogenetic trees were constructed using MEGA X software (version 10.2.6) with the maximum-likelihood method (1000 bootstraps) [56]. The genome sequences and annotations of *Arabidopsis*, rice, maize, and wheat were downloaded to analyze syntenic relations with barley. Syntenic relationships and gene duplications were analyzed using TBtools (version 2.119) [57,58].

### 4.4. Sequence, Promoter, and Variation Analyses

Conserved motifs of HvPLATZs were identified using MEME Suite 5.5.2 [59] with parameters set to classic mode, with zero or one occurrence per sequence (zoops). *Cis*-acting elements in the 2 kb upstream regions of *HvPLATZs* coding sequences were identified using PlantCARE (https://bioinformatics.psb.ugent.be/webtools/plantcare/html/, accessed on 17 April 2023) [60]. The pan-genome data [36] were obtained for variation analyses of 11 *HvPLATZ* genes.

### 4.5. Tissue-Specific Expression

Transcriptomic data (FPKM) of different tissues and stages were obtained from BARLEX (https://apex.ipk-gatersleben.de/apex/f?p=284:57::::::, accessed on 26 May 2023), normalized using a log_10_(FPKM + 1) transform, and visualized using TBtools [57].

### 4.6. Plant Growth and Abiotic Stress Treatments

Barley (cv. Golden promise) seeds were germinated in a 1/5 Hoagland solution for 6 d. The seedlings exhibiting uniform growth were transplanted into 5 L black barrels, with 18 plants per barrel. Eight days later, the seedlings were subjected to salt stress (200 mM NaCl), potassium deficiency (0.01 mM K^+^), and osmotic stress (20% PEG8000) in the background of a 1/5 Hoagland solution [61]. Plants growing in a 1/5 Hoagland solution were set as control. After treatments for 0.5 h, 1 h, 3 h, 6 h, 1 d, and 3 d, barley roots and leaves were collected for qRT-PCR. All samples were collected in three replicates, with two plants per replicate. During the entire growth period, seedlings were well aerated in a growth room and solutions were renewed every three days [61].

### 4.7. qRT-PCR

The MiniBEST Plant RNA Extraction Kit (9769, TaKaRa, Shiga, Japan) and PrimeScript RT Master Mix (RR036A, TaKaRa, Japan) were used for RNA extraction and cDNA synthesis, respectively. ChamQ Universal SYBR qPCR Master Mix (Q711, Vazyme, Nanjing, China) was used for qRT-PCR with an LC480 II (Roche, Basel, Switzerland), conducting two technical replicates. Relative gene expressions were calculated using the 2^−ΔΔCT^ method [62], with *actin* as the internal reference [58]. The primer sequences are listed in File S6.

### 4.8. Heterologous Expression of HvPLATZs in Yeast

The PrimeScript™ II 1st Strand cDNA Synthesis Kit (6210A, TaKaRa, Japan) was employed for synthesizing the 1st strand of cDNA. *HvPLATZ* genes were amplified with gene-specific primers (File S7) using Phanta Max Master Mix (P525, Vazyme, China). The pYES2-NTB was linearized using KpnI-HF (R3142V, New England Biolabs, Ipswich, MA, USA) and then recombined with *HvPLATZ* PCR products using the ClonExpress II One Step Cloning Kit (C112, Vazyme, China). The recombinant vectors were transformed into DH5α cells (DL1001, WEIDI, Shanghai, China). Single colonies were verified via PCR and sequencing (File S7), and recombinant vectors were extracted using the TaKaRa MiniBEST Plasmid Purification Kit v.4.0 (9760, TaKaRa, Japan).

Recombinant vectors were transformed into an INVSC1 strain using the LiAc transformation method. The transformed INVSC1 strains were subsequently cultured on SD-Ura medium for 2 d. PCR-verified single colonies were then resuspended in sterile water (OD = 0.6), serially diluted (10^0^, 10^−1^, and 10^−2^), and cultured at 30 °C on SG-Ura media with different concentrations of NaCl (0, 0.5, and 1.0 M) for 5 d. For the drought tolerance assay, single colonies were resuspended in sterile water (OD = 0.2) and shaken at 30 °C cultured on SG-Ura media containing various concentrations of PEG3350 (0, 30, 60, 90, and 120 mM) for 3 d. Yeast solutions were serially diluted (10^−1^, 10^−2^, and 10^−3^) and cultured at 30 °C on SG-Ura media for 4 d.

## 5. Conclusions

Eleven *HvPLATZ* genes were identified in barley and were classified into five clusters based on phylogeny. All *HvPLATZ* members possessed a typical PLATZ domain and exhibited conserved motif arrangements. Segmental duplication facilitated PLATZ family expansion in barley. *HvPLATZs* were core genes present in 20 genotypes of the barley pan-genome. The promoters of *HvPLATZs* contained a number of *cis*-acting elements involved in hormone and stress responses. The expression of *HvPLATZs* was influenced by abiotic stress and tissue type; furthermore, it varied over time. *HvPLATZ2* negatively regulated salt tolerance in yeast, whereas *HvPLATZ3/5/6/8/9/10/11* positively regulated salt tolerance. These findings advance our comprehension of the *PLATZ* family in barley and underpin further comprehensive functional characterization and breeding utilization of *HvPLATZs*.

## Figures and Tables

**Table 1:** Characteristics of 11 HvPLATZs.

Name	Gene ID	Length (aas)	Intron	MW (kDa)	pI	Instability Index	SL
HvPLATZ1	*HORVU.MOREX.r3.1HG0014730*	258	1	28.54	7.15	71.76	nucleus
HvPLATZ2	*HORVU.MOREX.r3.1HG0053060*	272	1	29.21	9.25	63.94	nucleus
HvPLATZ3	*HORVU.MOREX.r3.2HG0197200*	246	3	27.14	9.37	51.36	nucleus
HvPLATZ4	*HORVU.MOREX.r3.3HG0322980*	215	2	24.49	8.52	52.21	extracellular space
HvPLATZ5	*HORVU.MOREX.r3.6HG0568240*	237	3	27.19	8.67	60.13	nucleus
HvPLATZ6	*HORVU.MOREX.r3.6HG0571240*	240	3	26.24	8.72	55.73	nucleus
HvPLATZ7	*HORVU.MOREX.r3.6HG0574010*	215	2	24.08	7.95	48.26	nucleus
HvPLATZ8	*HORVU.MOREX.r3.6HG0605480*	249	3	26.85	9.44	56.24	nucleus
HvPLATZ9	*HORVU.MOREX.r3.7HG0690090*	212	2	22.74	8.88	48.06	nucleus
HvPLATZ10	*HORVU.MOREX.r3.7HG0723280*	241	3	26.75	9.27	58.83	nucleus
HvPLATZ11	*HORVU.MOREX.r3.7HG0738060*	254	3	27.87	8.69	59.8	nucleus

aas: amino acids; pI: isoelectric point; MW: molecular weight; SL: subcellular localization.

## Data Availability

Data is contained within the article and Supplementary Materials.

## Supplementary Materials

The supporting information can be downloaded at: https://www.mdpi.com/article/10.3390/ijms251810191/s1.
