# Supplementary material for: Identification and Functional Characterization of Abiotic Stress Tolerance-Related PLATZ Transcription Factor Family in Barley (Hordeum vulgare L.)
Source: Int J Mol Sci. 2024 Sep 23;25(18):10191. doi: 10.3390/ijms251810191 (PMC11432580; doi:10.3390/ijms251810191)
Supplement: Supplementary file 1 [file ijms-25-10191-s001.zip › File S5. Tissue expression patterns of HvPLATZs.pdf]

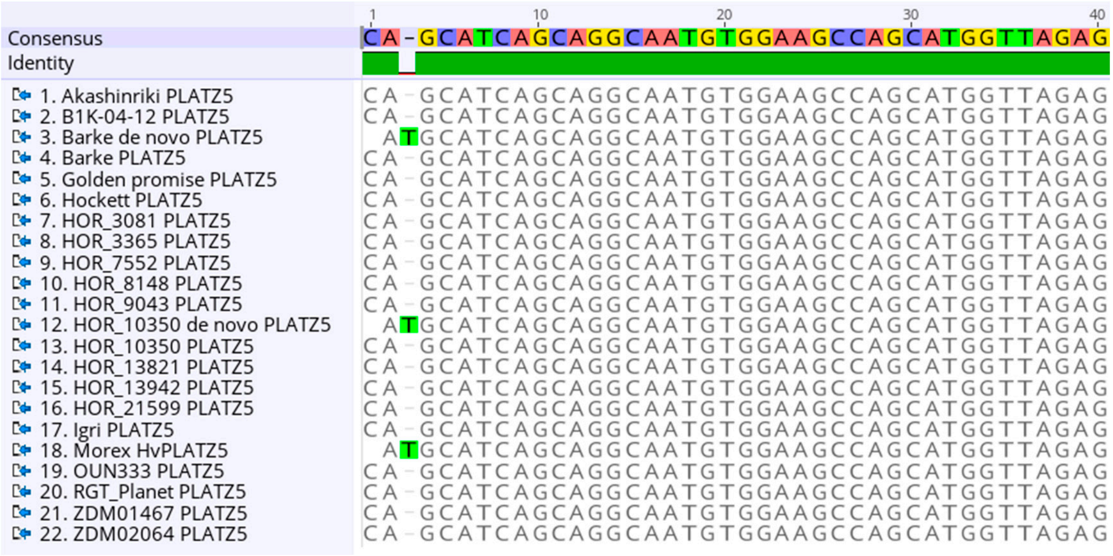

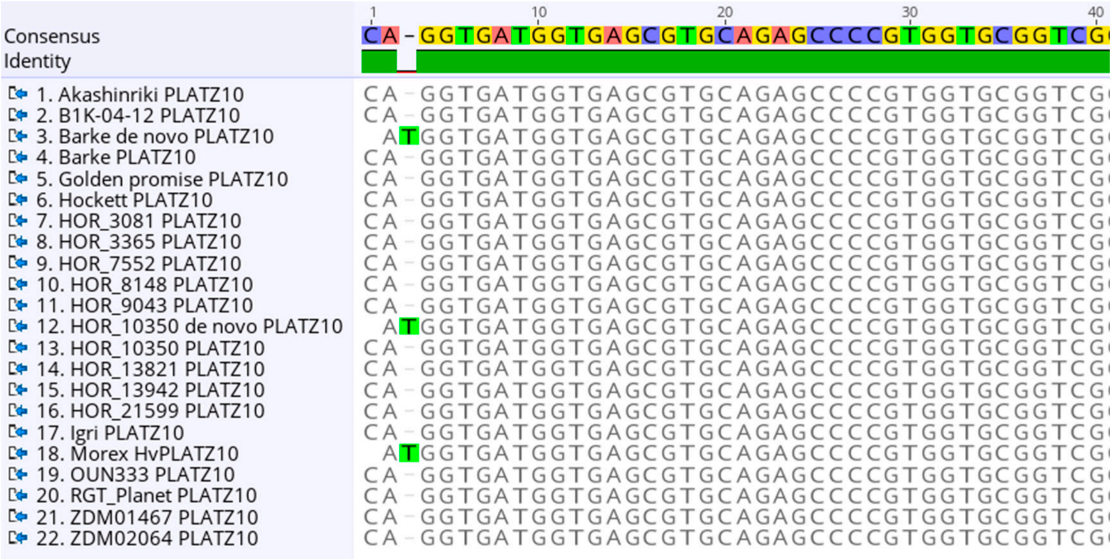

Coding sequence alignment of *HvPLATZ5* and *HvPLATZ10* from different genotypes and assemblies.
